# Selection of a suitable reference gene for quantitative gene expression in mouse lymph nodes after vaccination

**DOI:** 10.1186/s13104-017-3005-y

**Published:** 2017-12-06

**Authors:** Yung-Yi C. Mosley, Harm HogenEsch

**Affiliations:** Department of Comparative Pathobiology, College of Veterinary Medicine, Purdue University, 725 Harrison Street, West Lafayette, IN 47907 USA

**Keywords:** DTaP, Lymph nodes, Mouse, qPCR, Reference gene, *Ubc*

## Abstract

**Background:**

The quantification of gene expression is an important tool in the evaluation of the immune response to vaccines. Reliable reference genes for gene expression studies in mouse draining lymph nodes after vaccination have not been reported.

**Results:**

The utility of seven potential reference genes was investigated using commercially available Taq-man primer/probe mixes. Results were evaluated with RefFinder, a web-based program including multiple algorithm methods such as geNorm, NormFinder, BestKeeper and the comparative delta-Ct. Further assessment was done by applying the candidate reference genes in relative expression calculations with genes related to the magnitude and longevity of the humoral immune responses. The ubiquitin C gene, *Ubc,* was found to be the most reliable reference gene when validated with well-known genes that are expressed at relatively low levels after vaccination. The optimal time of sample collection varied depending on the function of the target genes.

**Conclusions:**

This study identified *Ubc* as the most reliable reference gene and provides useful information for studies examining immunological gene expression in the draining lymph nodes after vaccination in mice.

**Electronic supplementary material:**

The online version of this article (10.1186/s13104-017-3005-y) contains supplementary material, which is available to authorized users.

## Background

Quantitative real time PCR (qPCR) is an essential technique that has brought great advances in life sciences. Relative quantification of mRNA levels is widely used to determine the expression levels of target genes [1]. The accuracy of this assay relies heavily on the choice of reference genes for normalization [2, 3]. Ideal reference genes must have a relatively high but stable expression with minimal variation between individual samples and without disturbance from the experimental conditions [2, 3]. Commonly used reference genes such as β-actin (*Actb*) and glyceraldehyde-3-phosphate dehydrogenase (*Gapdh*) may not be suitable for every experimental condition [4, 5]. Thus, it is critical to select appropriate reference gene(s) for each circumstance including particular tissues and specific experimental designs [6, 7].

Upon vaccination, antigen presenting cells at the injection site take up vaccine antigens and actively migrate via a lymph vessel to the draining lymph nodes, followed by antigen processing and presentation to naïve T cells which initiates the adaptive immune responses to the vaccine antigens [8]. The activated antigen-specific T and B cells will interact, further differentiate and proliferate. Enormous changes in gene expression accompany the activation of the adaptive immune system. Several genes including tyrosine 3-monooxygenase/tryptophan 5-monooxygenase activation protein, zeta polypeptide (*Ywhaz*), ubiquitin C (*Ubc*), and *Actb* have been proposed as suitable reference genes for human lymph nodes with B cell lymphomas and non-neoplastic specimens [9]. On the other hand, while *Ubc* was identified as the most stable reference gene, *Actb* was recognized as an inappropriate reference genes in mouse lymphocytes purified from lymph nodes under normal condition [10]. Other genes including hypoxanthine phosphoribosyltransferase (*Hprt*), hydroxymethylbilane synthase (*Hmbs*), and TATA box-binding protein (*Tbp*) have been reported as suitable reference genes for lymphoid tissues in human, marmosets, goats and chickens [4, 11–14]. It is uncertain which reference gene would be best suited for analysis of gene expression in the draining lymph node by RT-qPCR following vaccination. Here, we examined the expression of the aforementioned genes and determined their suitability as reference genes for draining and non-draining lymph nodes in mice after vaccination with a diphtheria–tetanus–acellular pertussis vaccine (DTaP). The analysis included software tools such as geNorm, NormFinder, BestKeeper and the comparative delta-Ct method, and validation with genes that are known to be induced upon vaccination.

## Methods

### Animal studies

Thirty 5 week old female BALB/cJ mice (Jackson Laboratory, Bar Harbor, ME, USA) were housed at 3–4 mice/box in ventilated racks with a 12 h light/12 h dark–light cycle and ad lib access to water and chow. Mice were injected intramuscularly with 50 µl of DTaP vaccine, containing diphtheria toxoid, tetanus toxoid, pertussis toxin, filamentous hemagglutinin, and pertactin (Infanrix^®^, Glaxo Smith Kline, Rixensart, Belgium) at 6, 8, and 12 weeks of age in alternating hind legs. Mice were euthanized by CO_2_ inhalation and cervical dislocation for sample collection. The protocol was approved by Purdue University Animal Care and Use Committee.

### RNA extraction

Axillary (non-draining) and iliac (draining) lymph nodes [15–17] were collected at 1 and 7 days after the first and third vaccination (V1D1, V1D7, V3D1 and V3D7, respectively) and stored in RNA*later* ^®^ (Life technologies, Carlsbad, CA, USA) at 4 °C for at least 24 h before RNA extraction. Fat tissue around lymph nodes was removed with a fine tip forceps and 30G needle under a dissecting microscope. Excess RNAlater^®^ was blotted away and the tissue was homogenized with a mortar and pestle in the presence of liquid nitrogen. The total RNA from homogenized tissue was extracted by Quick-RNA™ MiniPrep Plus (Zymo Research, Irvine, CA, USA) following manufacturer’s instruction with proteinase K and DNase I digestion. RNA concentration and purity were checked with a NanoDrop spectrophotometer (Thermo Scientific, Wilmington, DE, USA).

### Reverse transcription

Total RNA of 1 µg was reverse-transcribed with iScript™ cDNA Synthesis Kit (Bio-Rad, Hercules, CA, USA) in a total volume of 20 µl which contains a blend of oligo dT/random hexamer primers. The synthesis of cDNA was conducted in Mastercycler^®^ pro (Eppendorf, Hamburg, Germany) with the following protocol: priming at 25 °C for 5 min, reverse transcription at 42 °C for 30 min and inactivation at 85 °C for 5 min. The interference of genomic DNA carryover in RNA samples was tested using no-RT control supermix included in the cDNA synthesis kit.

### Quantitative real time PCR

Real time qPCR was performed in a total volume of 10 µl using iTaq™ Universal Probes Supermix (2× concentrated master mix) (Bio-Rad) and primer/probe sets from Taqman^®^ Gene Express Assay (Applied Biosystems, Waltham, MA, USA) with 5′-FAM TaqMan^®^ MGB probe and 3′-nonfluorescent quencher for genes including *Hprt* (Mm03024075_m1), *Hmbs* (Mm01143545_m1), *Gapdh* (Mm99999915_g1), *Ubc* (Mm01201237_m1), β-actin (*Actb,* Mm00607939_s1), *Tbp* (Mm01277045_m1), *Ywhaz* (Mm03950126_s1), interferon regulatory factor 4 (*Irf4*, Mm00516431_m1), interleukin 4 (*Il4,* Mm00445259_m1), zinc finger and BTB domain-containing protein 20 (*Zbtb20*, Mm00457765_m1) and tumor necrosis factor receptor superfamily member 17 (*Tnfrsf17*, Mm00495683_m1). All samples were prepared in duplicate in strips with clear cap (VWR, Radnor, PA, USA). No-RT and non-template controls were included for each qPCR run in Mastercycler^®^ RealPlex4 (Eppendorf) with cycle profile as denaturing at 95 °C for 30 s and 40 cycles at 95 °C for 15 s, 60 °C for 60 s.

### Data and statistical analysis

Mean values of the quantification cycle (Cq) [6] from duplicate reactions were used to determine the stability of each reference gene by RefFinder (http://150.216.56.64/referencegene.php or http://leonxie.esy.es/RefFinder/) [18], a web-based tool for comparing and ranking reference genes with four computational programs (geNorm [19], NormFinder [20], BestKeeper [21] and the comparative delta-Ct method [22]). Reference genes were also evaluated with the relative gene expression levels calculated by the ΔΔCq method [23] with fold changes between immunized and age-matched naïve mice.

Data are presented as mean ± SD unless indicated otherwise. The statistical significance of differences between groups was determined by Student’s t test or one-way ANOVA followed by Tukey’s multiple comparison test using GraphPad Prism 6 (GraphPad software, La Jolla, CA, USA). For relative gene expression, ΔΔCq values were used for statistical analysis.

## Results

### RNA quality and RT controls

The draining lymph nodes in vaccinated mice were larger than the non-draining lymph nodes and lymph nodes in unvaccinated mice and this was reflected in the RNA concentrations. The average RNA concentrations of the non-draining axillary lymph nodes (aLN) from vaccinated (177.94 ± 59.50 ng/µl; range 84.51–306.71) and naïve mice (177.57 ± 83.21 ng/µl; range 78.24–335.33) were similar. On the other hand, the RNA concentration of the draining iliac lymph nodes (iLN) was significantly larger (*P* = 0.0017) in vaccinated mice (371.49 ± 232.27 ng/µl; range 109.07–930.85) than naïve mice (111.64 ± 30.39 ng/µl; range 51.28–162.59). For vaccinated mice, the total RNA concentrations obtained from the iLN were significantly higher than those from aLN (*P* = 0.0009). The RNA of one mouse from the V1D1 group had a very low RNA concentration (22.83 ng/µl) and A_260/280_ ratio (1.61) and was removed from further experiments. The A_260/280_ ratio of RNA samples among all axillary and iLNs were within a narrow variation with a mean of 1.94 ± 0.04 (range 1.82–2.05).

**Fig. 1:**
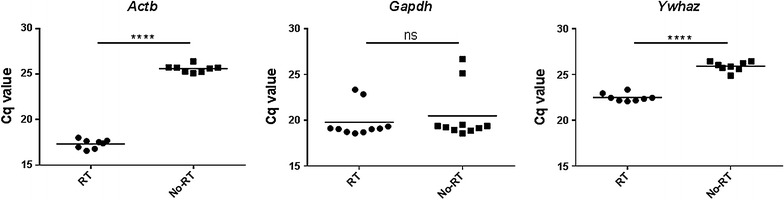
Comparison of Cq values from RT and No-RT samples. Three of the seven primer/probe sets detected signals from No-RT controls (n = 8–10) in qPCR including *Actb*, *Gapdh* and *Ywhaz*. When the Cq values of No-RT controls were compared to those of RT samples (RNA samples went through reverse transcription step), significant larger values of No-RT controls was observed for *Actb* and *Ywhaz*, but not for *Gapdh*

Although DNase was used during the RNA extraction process, three of the primer/probe sets detected signals from No-RT controls in qPCR including *Actb*, *Gapdh* and *Ywhaz*. While the Cq values of No-RT controls were significantly larger than those of cDNA samples for *Actb* and *Ywhaz*, those of *Gapdh* showed no difference (Fig. 1). There was no signal detected from non-template controls.

### Identification of the optimal reference gene by RefFinder software

**Fig. 2:**
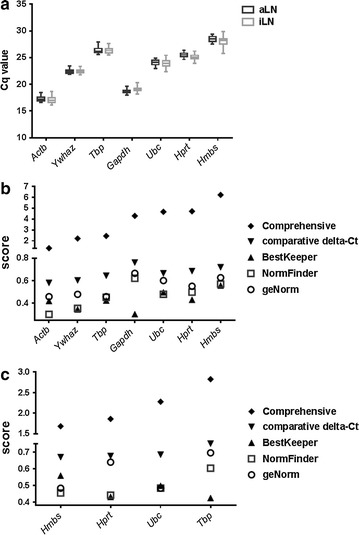
Evaluation of reference gene expression. **a** Gene expression by RT-qPCR. Average Cq values from duplicate qPCR reactions of each aLN (n = 30) and iLN sample (n = 29) were used for data analysis. The box indicates 25–75% with median shown in the box and the whiskers represent the minimum to maximum range. **b** Scores determined by RefFinder including the comprehensive stability, geNorm, NormFinder, BestKeeper and comparative delta-Ct method are shown for 7 reference genes. **c** Scores determined by RefFinder including the comprehensive stability, geNorm, NormFinder, BestKeeper and comparative delta-Ct method are shown for 4 reference genes

Lower Cq values among the seven reference genes were observed from *Actb*, *Gapdh* and *Ywhaz* (Fig. 2a, Additional file 1: Tables S1, S2). Although this suggests abundant expression of these three genes, this may be confounded by contributions from genomic DNA and/or non-specific signals. Among the rest of the four reference genes, *Ubc* had the lowest Cq value (24.00 ± 0.62) and *Hprt* had the smallest sample variation when Cq values were averaged from total LNs (25.31 ± 0.54).

RefFinder assesses potential reference genes based on four computational programs: geNorm, NormFinder, BestKeeper and the comparative delta-Ct method, and provides a comprehensive score to rank the stability of the reference genes across experimental groups (higher stability with lower scores). When all seven genes were included in the analysis, RefFinder identified *Actb* as the most stable gene (by comprehensive score), followed by *Ywhaz* and *Tbp* (Fig. 2b and Additional file 1: Table S3). However, genomic DNA and nonspecific signals in *Actb*, *Gapdh* and *Ywhaz* samples could confound the stability assessment. After eliminating these genes, the comprehensive score recognized *Hmbs* as the most stable gene (Fig. 2c and Additional file 1: Table S4). However, the stability scores given by the four programs were not consistent. For example, while NormFinder gave the lowest score to *Hprt*, geNorm gave the lowest score to both *Hmbs* and *Ubc* (Fig. 2c and Additional file 1: Table S4).

### Evaluation of the optimal reference gene by *Irf4*, *Zbtb20*, *Tnfrsf17* and *Il4* expression

The suitability of the reference genes was further evaluated by ΔΔCq method with target genes that are known to be expressed after vaccinations. The expression of *Tnfrsf17* is associated with antibody magnitude following immunization with a live attenuated yellow fever vaccine, trivalent inactivated influenza vaccine and polysaccharide-protein conjugate vaccine for *Neisseria meningitidis,* and is a potential biomarker for predicting the antibody response at 2–3 months after vaccination [24–26]. Since DTaP is an aluminum-adjuvanted vaccine which is known to induce a Th2-biased immune response [27, 28], we also examined the expression of IL-4. In addition, IRF4 and ZBTB20 have been identified as crucial transcription factors for long term survival of plasma cells with aluminum-adjuvanted antigens [29, 30]. Although their induced expression levels are expected to be relatively low, we included these two genes as positive controls following DTaP vaccination.

**Fig. 3:**
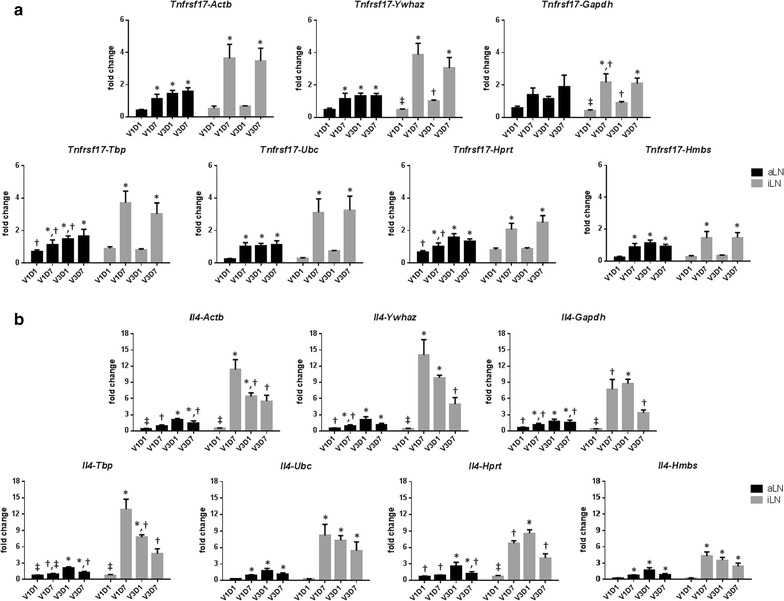
Effect of reference genes on the calculated expression of *Tnfrsf17* and *Il4*. The relative expression of *Tnfrsf17* (**a**) and *Il4* (**b**) in aLN and iLN were normalized with *Actb*, *Ywhaz*, *Gapdh*, *Tbp*, *Ubc*, *Hprt* or *Hmbs* by RT-qPCR after 1 and 7 days of the 1st and 3rd vaccination (V1D1, V1D7, V3D1 and V3D7, respectively). Data are presented as mean + SEM. Comparison of ΔΔCq values by one-way ANOVA and Tukey’s multiple comparison test was performed. Experimental groups identified with different symbols were significantly different from each other (*P* < 0.05)

The reference genes were evaluated by using their Cq values to calculate the relative expression level for each of the target genes. A significantly higher expression of *Tnfrsf17* was observed at 7 days after the 1st and 3rd vaccination but not at 1 day after vaccination with all seven reference genes (Fig. 3a). The expression of *Il4* was increased at 7 days after the 1st vaccination and the level was not enhanced after the 3rd vaccination although the calculated fold-change varied among different reference genes (Fig. 3b). Overall, the use of these seven reference genes yielded similar patterns of relative gene expression for *Tnfrsf17* and *Il4* (Fig. 3).

**Fig. 4:**
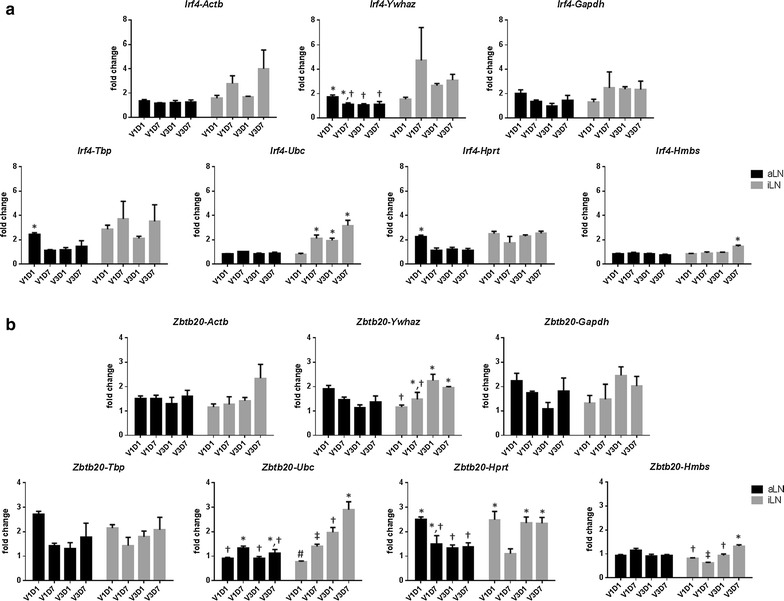
Effect of reference genes on the calculated expression of *Irf4* and *Zbtb20*.The relative expression of *Irf4* (**a**) and *Zbtb20* (**b**) in aLN and iLN were normalized with *Actb*, *Ywhaz*, *Gapdh*, *Tbp*, *Ubc*, *Hprt* or *Hmbs* by RT-qPCR after 1 and 7 days of the 1st and 3rd vaccination (V1D1, V1D7, V3D1 and V3D7, respectively). Data are presented as mean + SEM. Comparison of ΔΔCq values by one-way ANOVA and Tukey’s multiple comparison test was performed. Experimental groups identified with different symbols were significantly different from each other (*P* < 0.05)

However, the variation caused by using a different reference gene became evident when the induced expression levels of target genes was lower such as for *Irf4* and *Zbtb20* (Fig. 4). Among the seven reference genes, *Ubc* consistently delivered low background expression in non-draining aLN and more distinct expression of *Irf4* and *Zbtb20* in draining iLNs (Fig. 4). The use of *Hmbs* yielded similar pattern but lower expression levels, whereas *Actb*, *Ywhaz*, *Gapdh*, *Hprt* and *Tbp* failed to show the low grade expression patterns (Fig. 4).

## Discussion

The current study was carried out to search for suitable reference genes for gene expression studies in mouse draining lymph nodes after DTaP vaccination. The samples collected after the first vaccination (V1D1 and V1D7) were considered priming responses whereas those collected after the third vaccination (V3D1 and V3D7) were mainly a memory response which reacts quicker and stronger compared to the primary response.

As expected, the total RNA concentrations obtained from the draining lymph nodes were significantly larger than those from non-draining lymph nodes from vaccinated mice and the iLN and aLN of naïve mice. For the genes whose No-RT controls showed signals, the Cq values of No-RT were significantly larger than those of cDNA samples for *Actb* and *Ywhaz*, and those of *Gapdh* showed no difference. It indicates the signals of No-RT controls from *Actb* and *Ywhaz* are derived from residual genomic DNA and those of *Gapdh* might be non-specific amplification.

The expression of *Tnfrsf17* was significantly higher at 7 days after the 1st and 3rd vaccination but not at 1 day after vaccination with all seven reference genes. This result is consistent with previous studies that *Tnfrsf17* expression is associated with antibody magnitude after 7 days but not at 1 or 3 days after vaccination [24–26]. TNFRSF17, also known as B cell maturation antigen (BCMA), is expressed in terminally-differentiated B cells and is one of the three receptors for B cell activating factor (BAFF). While loss of the BCMA receptor has no impact on B cell development and the generation of mature peripheral B cells, the BCMA signaling pathway is important for the survival of plasma cells [31].

The computer programs used in this study identified *Hmbs* as the most stable gene when *Actb* and *Ywhaz*, and *Gapdh* were not included in the analysis. However the ΔΔCq method using gene expression of transcription factors confirmed *Ubc* performed better than *Hmbs* as a reference gene. These results also suggest that geNorm provides more relevant assessment than the other computational programs used in this study. In addition to the uniformity of gene expression, ideal reference genes should have similar expression levels with the target genes [2]. Although the mean Cq values of *Actb* was within the moderate expression range of 15–30 Cq [32], it was the lowest (17 ± 0.53) among the seven reference genes and created the largest ΔCq values with the Cq values from the targets genes, i.e., the average Cq values of target genes ranges from 26.63–30.18. The Cq values of *Actb* might lose its sensitivity to reflect the difference in sample loading or RNA extraction due to its abundant expression.

The suitable reference gene identified in this study was *Ubc*, which encodes the protein polyubiquitin C. Polyubiquitin C is one of the precursors for ubiquitin (Ub) that has nine tandem repeats of Ub and is cleaved by deubiquitinating enzymes to release functional monomeric Ub units [33]. Ubiquitination regulates all aspects of cell biology, including protein degradation, cell signaling, cell cycle, gene expression, intracellular trafficking and the DNA damage response [34]. Although ubiquitination plays an important role in the regulation of immune responses such as dendritic cell maturation and T cells activation and differentiation [35], the expression of *Ubc* was found to remain stable following vaccination in the current study.

## Conclusion

The choice of reference gene can markedly affect the interpretation of gene expression especially for those genes with low expression levels. Here, we identified *Ubc* as the ideal reference gene for gene expression analysis in mouse lymph nodes following DTaP vaccination. Although the commercial Taqman primer/probe sets are generally well-designed, careful evaluation for each application is recommended.

## Additional file

**Additional file 1.** Selection of a suitable reference gene for quantitative gene expression in mouse lymph nodes after vaccination.

## Abbreviations

qPCR: quantitative real time PCR
*Actb*: β-actin
*Gapdh*: glyceraldehyde-3-phosphate dehydrogenase
*Ywhaz*: tyrosine 3-monooxygenase/tryptophan 5-monooxygenase activation protein, zeta polypeptide
*Ubc*: ubiquitin C
*Hprt*: hypoxanthine phosphoribosyltransferase
*Hmbs*: hydroxymethylbilane synthase
*Tbp*: TATA box-binding protein
DTaP: diphtheria–tetanus–acellular pertussis vaccine
V1D1: one day after the 1st vaccination
V1D7: seven days after the 1st vaccination
V3D1: one day after the 3rd vaccination
V3D7: seven days after the 3rd vaccination
*Irf4*: interferon regulatory factor 4
*Il4*: interleukin 4
*Zbtb20*: zinc finger and BTB domain-containing protein 20
*Tnfrsf17*: tumor necrosis factor receptor superfamily member 17
aLN: axillary lymph node
iLN: iliac lymph node
Cq: quantification cycle
BCMA: B cell maturation antigen
BAFF: B cell activating factor
Ub: ubiquitin

## References

[1] VanGuilder HD, Vrana KE, Freeman WM (2008). Twenty-five years of quantitative PCR for gene expression analysis. Biotechniques.

[2] Kozera B, Rapacz M (2013). Reference genes in real-time PCR. J Appl Genet.

[3] Thellin O, Zorzi W, Lakaye B, De Borman B, Coumans B, Hennen G, Grisar T, Igout A, Heinen E (1999). Housekeeping genes as internal standards: use and limits. J Biotechnol.

[4] Dheda K, Huggett JF, Bustin SA, Johnson MA, Rook G, Zumla A (2004). Validation of housekeeping genes for normalizing RNA expression in real-time PCR. Biotechniques.

[5] Hruz T, Wyss M, Docquier M, Pfaffl MW, Masanetz S, Borghi L, Verbrugghe P, Kalaydjieva L, Bleuler S, Laule O (2011). RefGenes: identification of reliable and condition specific reference genes for RT-qPCR data normalization. BMC Genomics.

[6] Bustin SA, Benes V, Garson JA, Hellemans J, Huggett J, Kubista M, Mueller R, Nolan T, Pfaffl MW, Shipley GL (2009). The MIQE guidelines: minimum information for publication of quantitative real-time PCR experiments. Clin Chem.

[7] Jeong JK, Kang MH, Gurunathan S, Cho SG, Park C, Seo HG, Kim JH (2014). Evaluation of reference genes in mouse preimplantation embryos for gene expression studies using real-time quantitative RT-PCR (RT-qPCR). BMC Res Notes.

[8] Parham P (2015). The immune system.

[9] Potashnikova D, Gladkikh A, Vorobjev IA (2015). Selection of superior reference genes’ combination for quantitative real-time PCR in B-cell lymphomas. Ann Clin Lab Sci.

[10] Albershardt TC, Iritani BM, Ruddell A (2012). Evaluation of reference genes for quantitative PCR analysis of mouse lymphocytes. J Immunol Methods.

[11] Zhang Y, Zhang XD, Liu X, Li YS, Ding JP, Zhang XR, Zhang YH (2013). Reference gene screening for analyzing gene expression across goat tissue. Asian–Australas J Anim Sci.

[12] Ledderose C, Heyn J, Limbeck E, Kreth S (2011). Selection of reliable reference genes for quantitative real-time PCR in human T cells and neutrophils. BMC Res Notes.

[13] Tsaur I, Renninger M, Hennenlotter J, Oppermann E, Munz M, Kuehs U, Stenzl A, Schilling D (2013). Reliable housekeeping gene combination for quantitative PCR of lymph nodes in patients with prostate cancer. Anticancer Res.

[14] Fujii Y, Kitaura K, Matsutani T, Shirai K, Suzuki S, Takasaki T, Kumagai K, Kametani Y, Shiina T, Takabayashi S (2013). Immune-related gene expression profile in laboratory common marmosets assessed by an accurate quantitative real-time PCR using selected reference genes. PLoS ONE.

[15] Van den Broeck W, Derore A, Simoens P (2006). Anatomy and nomenclature of murine lymph nodes: descriptive study and nomenclatory standardization in BALB/cAnNCrl mice. J Immunol Methods.

[16] Langlet C, Tamoutounour S, Henri S, Luche H, Ardouin L, Grégoire C, Malissen B, Guilliams M (2012). CD64 expression distinguishes monocyte-derived and conventional dendritic cells and reveals their distinct role during intramuscular immunization. J Immunol.

[17] Lu F, Mosley Y-YC, Rosales RJ, Carmichael BE, Elesela S, Yao Y, HogenEsch H (2017). Alpha-D-glucan nanoparticulate adjuvant induces a transient inflammatory response at the injection site and targets antigen to migratory dendritic cells. npj Vaccines.

[18] Xie F, Xiao P, Chen D, Xu L, Zhang B. miRDeepFinder: a miRNA analysis tool for deep sequencing of plant small RNAs. Plant Mol Biol 2012. 10.1007/s11103-012-9885-2 22290409

[19] Vandesompele J, De Preter K, Pattyn F, Poppe B, Van Roy N, De Paepe A, Speleman F (2002). Accurate normalization of real-time quantitative RT-PCR data by geometric averaging of multiple internal control genes. Genome Biol.

[20] Andersen CL, Jensen JL, Ørntoft TF (2004). Normalization of real-time quantitative reverse transcription-PCR data: a model-based variance estimation approach to identify genes suited for normalization, applied to bladder and colon cancer data sets. Cancer Res.

[21] Pfaffl MW, Tichopad A, Prgomet C, Neuvians TP (2004). Determination of stable housekeeping genes, differentially regulated target genes and sample integrity: bestkeeper–excel-based tool using pair-wise correlations. Biotechnol Lett.

[22] Silver N, Best S, Jiang J, Thein SL (2006). Selection of housekeeping genes for gene expression studies in human reticulocytes using real-time PCR. BMC Mol Biol.

[23] Livak KJ, Schmittgen TD (2001). Analysis of relative gene expression data using real-time quantitative PCR and the 2(-Delta Delta C(T)) Method. Methods.

[24] Querec TD, Akondy RS, Lee EK, Cao W, Nakaya HI, Teuwen D, Pirani A, Gernert K, Deng J, Marzolf B (2009). Systems biology approach predicts immunogenicity of the yellow fever vaccine in humans. Nat Immunol.

[25] Li S, Rouphael N, Duraisingham S, Romero-Steiner S, Presnell S, Davis C, Schmidt DS, Johnson SE, Milton A, Rajam G (2014). Molecular signatures of antibody responses derived from a systems biology study of five human vaccines. Nat Immunol.

[26] Nakaya HI, Wrammert J, Lee EK, Racioppi L, Marie-Kunze S, Haining WN, Means AR, Kasturi SP, Khan N, Li GM (2011). Systems biology of vaccination for seasonal influenza in humans. Nat Immunol.

[27] Ross PJ, Sutton CE, Higgins S, Allen AC, Walsh K, Misiak A, Lavelle EC, McLoughlin RM, Mills KH (2013). Relative contribution of Th1 and Th17 cells in adaptive immunity to bordetella pertussis: towards the rational design of an improved acellular pertussis vaccine. PLoS Pathog.

[28] HogenEsch H (2002). Mechanisms of stimulation of the immune response by aluminum adjuvants. Vaccine.

[29] Chevrier S, Emslie D, Shi W, Kratina T, Wellard C, Karnowski A, Erikci E, Smyth GK, Chowdhury K, Tarlinton D (2014). The BTB-ZF transcription factor Zbtb20 is driven by Irf4 to promote plasma cell differentiation and longevity. J Exp Med.

[30] Wang Y, Bhattacharya D (2014). Adjuvant-specific regulation of long-term antibody responses by ZBTB20. J Exp Med.

[31] Coquery CM, Erickson LD (2012). Regulatory roles of the tumor necrosis factor receptor BCMA. Crit Rev Immunol.

[32] Lland H, Hertzberg M, Marlton P (2006). Myeloid Leukemia Methods and Protocols.

[33] Kimura Y, Tanaka K (2010). Regulatory mechanisms involved in the control of ubiquitin homeostasis. J Biochem.

[34] Swatek KN, Komander D (2016). Ubiquitin modifications. Cell Res.

[35] Hu H, Sun SC (2016). Ubiquitin signaling in immune responses. Cell Res.

