# Supplementary material for: Selection of a suitable reference gene for quantitative gene expression in mouse lymph nodes after vaccination
Source: BMC Res Notes. 2017 Dec 6;10:689. doi: 10.1186/s13104-017-3005-y (PMC5718095; doi:10.1186/s13104-017-3005-y)
Supplement: Supplementary file 1 — Additional file 1. Selection of a suitable reference gene for quantitative gene expression in mouse lymph nodes after vaccination. [file 13104_2017_3005_MOESM1_ESM.docx]

**Additional information**

**Selection of a suitable reference gene for quantitative gene expression in mouse lymph nodes after vaccination**

Yung-Yi C. Mosley*, Harm HogenEsch

Department of Comparative Pathobiology, College of Veterinary Medicine, Purdue University, West Lafayette, Indiana, USA

Table S1. Cq values of each gene in aLN samples

|  | ***Hprt*** | ***Ubc*** | ***Gapdh*** | ***Hmbs*** | ***Actb*** | ***Tbp*** | ***Ywhaz*** | ***Tnfrsf17*** | ***Il4*** | ***Irf4*** | ***Zbtb20*** |
| --- | --- | --- | --- | --- | --- | --- | --- | --- | --- | --- | --- |
| aLN C1-1 | 25.21 | 23.89 | 18.39 | 28.59 | 17.34 | 25.85 | 22.05 | 26.58 | 30.26 | 27.16 | 26.89 |
| aLN C1-2 | 25.62 | 24.48 | 18.30 | 29.15 | 17.53 | 26.34 | 22.48 | 27.74 | 32.63 | 27.65 | 27.54 |
| aLN C1-3 | 25.07 | 24.63 | 18.57 | 28.96 | 17.18 | 25.96 | 22.28 | 27.43 | 30.53 | 27.50 | 27.79 |
| aLN C1-4 | 25.90 | 24.85 | 17.96 | 29.37 | 17.51 | 25.98 | 22.32 | 28.19 | 31.10 | 27.84 | 28.02 |
| aLN C1-5 | 25.40 | 24.06 | 18.48 | 28.62 | 16.72 | 25.56 | 21.94 | 27.68 | 31.27 | 27.47 | 27.20 |
| aLN C2-1 | 25.54 | 24.55 | 18.94 | 28.80 | 17.29 | 26.74 | 22.56 | 27.38 | 31.51 | 27.07 | 26.65 |
| aLN C2-2 | 25.55 | 24.12 | 19.05 | 28.33 | 16.80 | 26.15 | 21.94 | 27.08 | 31.04 | 26.78 | 26.57 |
| aLN C2-3 | 24.65 | 24.07 | 19.11 | 28.15 | 16.58 | 25.84 | 22.95 | 26.65 | 31.21 | 27.08 | 26.31 |
| aLN C2-4 | 25.45 | 24.27 | 18.80 | 28.54 | 17.10 | 25.84 | 22.15 | 27.68 | 32.46 | 27.13 | 27.09 |
| aLN C2-5 | 25.19 | 24.69 | 18.68 | 29.43 | 18.28 | 27.39 | 23.45 | 27.11 | 31.65 | 27.84 | 27.89 |
| aLN V1D1-1 | 25.74 | 23.12 | 18.34 | 27.87 | 16.68 | 26.33 | 22.22 | 28.08 | 32.05 | 26.52 | 26.64 |
| aLN V1D1-2 | 26.30 | 23.70 | 18.14 | 28.33 | 16.97 | 26.70 | 22.36 | 28.40 | 32.33 | 26.99 | 26.85 |
| aLN V1D1-3 | 26.04 | 23.57 | 18.40 | 28.02 | 16.92 | 26.70 | 22.58 | 30.41 | 32.75 | 26.98 | 26.69 |
| aLN V1D1-4 | 25.80 | 23.38 | 18.67 | 28.12 | 17.11 | 26.52 | 22.16 | 28.83 | 31.83 | 27.09 | 26.70 |
| aLN V1D1-5 | 25.73 | 23.29 | 18.86 | 27.67 | 17.14 | 26.30 | 22.00 | 28.34 | 31.53 | 26.73 | 26.51 |
| aLN V1D1-6 | 25.42 | 22.94 | 18.68 | 27.55 | 16.68 | 26.18 | 22.04 | 28.02 | 32.62 | 26.22 | 26.05 |
| aLN V1D7-1 | 25.40 | 24.40 | 18.76 | 29.03 | 17.23 | 26.10 | 22.43 | 27.63 | 31.65 | 27.46 | 27.23 |
| aLN V1D7-2 | 26.40 | 24.25 | 18.74 | 28.85 | 17.66 | 26.24 | 22.59 | 28.33 | 32.38 | 27.55 | 26.94 |
| aLN V1D7-3 | 25.15 | 24.22 | 18.84 | 28.55 | 17.25 | 26.01 | 22.39 | 26.40 | 30.59 | 27.20 | 27.20 |
| aLN V1D7-4 | 25.51 | 24.63 | 18.70 | 28.78 | 17.64 | 26.21 | 22.35 | 27.88 | 31.35 | 27.73 | 27.18 |
| aLN V1D7-5 | 25.15 | 24.49 | 18.71 | 28.49 | 17.41 | 25.73 | 22.08 | 28.01 | 31.50 | 27.66 | 26.96 |
| aLN V3D1-1 | 24.88 | 23.62 | 18.85 | 27.66 | 16.88 | 25.92 | 21.85 | 26.43 | 29.92 | 26.54 | 26.04 |
| aLN V3D1-2 | 24.79 | 23.59 | 18.81 | 27.77 | 16.94 | 26.15 | 22.03 | 26.59 | 30.90 | 26.58 | 26.14 |
| aLN V3D1-3 | 25.98 | 24.16 | 18.68 | 28.08 | 17.18 | 26.14 | 23.02 | 26.82 | 30.15 | 27.04 | 26.74 |
| aLN V3D1-4 | 26.24 | 24.83 | 18.35 | 29.37 | 17.29 | 26.80 | 23.20 | 27.01 | 30.54 | 27.98 | 27.86 |
| aLN V3D1-5 | 25.75 | 24.01 | 18.84 | 29.02 | 18.44 | 27.66 | 23.09 | 27.27 | 31.98 | 27.59 | 27.08 |
| aLN V3D7-1 | 24.71 | 23.83 | 19.62 | 27.70 | 16.74 | 25.97 | 21.92 | 25.92 | 31.24 | 26.50 | 25.93 |
| aLN V3D7-2 | 26.25 | 24.59 | 18.58 | 28.94 | 17.55 | 26.86 | 23.36 | 27.58 | 31.47 | 27.70 | 27.21 |
| aLN V3D7-3 | 24.84 | 24.38 | 19.49 | 28.01 | 17.84 | 26.01 | 22.07 | 26.77 | 30.80 | 27.29 | 26.48 |
| aLN V3D7-4 | 25.74 | 23.72 | 19.11 | 28.19 | 17.86 | 27.89 | 23.34 | 27.20 | 32.40 | 27.15 | 26.60 |

.

Table S2. Cq values of each gene in iLN samples

|  | ***Hprt*** | ***Ubc*** | ***Gapdh*** | ***Hmbs*** | ***Actb*** | ***Tbp*** | ***Ywhaz*** | ***Tnfrsf17*** | ***Il4*** | ***Irf4*** | ***Zbtb20*** |
| --- | --- | --- | --- | --- | --- | --- | --- | --- | --- | --- | --- |
| iLN C1-1 | 24.92 | 24.45 | 18.42 | 28.84 | 17.23 | 25.83 | 22.33 | 26.71 | 28.88 | 27.39 | 27.47 |
| iLN C1-2 | 24.46 | 23.92 | 19.53 | 28.26 | 16.68 | 25.52 | 21.96 | 26.74 | 30.31 | 27.12 | 26.62 |
| iLN C1-3 | 25.13 | 23.91 | 19.35 | 28.34 | 16.52 | 25.89 | 22.01 | 27.28 | 30.44 | 27.24 | 26.43 |
| iLN C1-4 | 24.90 | 24.06 | 19.05 | 28.78 | 16.89 | 25.52 | 22.18 | 26.99 | 30.18 | 27.62 | 26.69 |
| iLN C1-5 | 25.11 | 23.43 | 19.38 | 28.20 | 16.59 | 25.58 | 21.80 | 26.49 | 29.66 | 26.48 | 26.01 |
| iLN C2-1 | 25.30 | 24.33 | 20.28 | 29.25 | 17.23 | 26.24 | 22.30 | 26.91 | 30.00 | 27.32 | 27.65 |
| iLN C2-2 | 25.76 | 25.43 | 18.74 | 29.92 | 17.64 | 26.70 | 22.59 | 26.51 | 30.36 | 28.38 | 28.12 |
| iLN C2-3 | 24.70 | 23.94 | 19.34 | 28.73 | 16.99 | 25.92 | 22.08 | 25.88 | 30.11 | 26.83 | 26.99 |
| iLN C2-4 | 25.02 | 23.61 | 19.18 | 28.48 | 17.90 | 27.42 | 22.14 | 26.38 | 29.97 | 26.41 | 26.42 |
| iLN C2-5 | 25.40 | 24.16 | 18.84 | 28.85 | 17.47 | 26.83 | 23.37 | 27.01 | 30.38 | 26.75 | 26.97 |
| iLN V1D1-1 | 25.49 | 22.37 | 19.23 | 27.04 | 16.32 | 26.43 | 21.95 | 27.65 | 31.25 | 26.31 | 25.47 |
| iLN V1D1-2 | 26.22 | 23.34 | 18.88 | 28.00 | 16.63 | 26.55 | 22.19 | 28.08 | 31.02 | 27.18 | 26.50 |
| iLN V1D1-3 | 25.62 | 23.65 | 18.85 | 28.14 | 17.59 | 26.73 | 22.03 | 27.51 | 31.06 | 26.84 | 26.72 |
| iLN V1D1-4 | 25.49 | 23.18 | 18.62 | 27.72 | 16.79 | 26.68 | 22.25 | 28.34 | 31.87 | 26.68 | 26.33 |
| iLN V1D1-5 | 25.40 | 22.75 | 18.85 | 27.32 | 16.63 | 26.51 | 21.97 | 28.23 | 30.67 | 26.03 | 25.60 |
| iLN V1D7-1 | 25.48 | 24.92 | 18.17 | 28.58 | 17.45 | 26.27 | 22.52 | 26.09 | 27.42 | 27.55 | 27.47 |
| iLN V1D7-2 | 23.92 | 23.48 | 18.92 | 26.90 | 16.68 | 25.67 | 22.35 | 24.80 | 26.06 | 26.27 | 25.82 |
| iLN V1D7-3 | 24.46 | 24.47 | 18.86 | 27.87 | 17.24 | 25.83 | 22.61 | 24.82 | 26.85 | 26.36 | 26.52 |
| iLN V1D7-4 | 24.77 | 24.37 | 18.66 | 27.58 | 18.02 | 27.03 | 22.47 | 25.70 | 27.04 | 26.31 | 26.35 |
| iLN V1D7-5 | 24.09 | 22.72 | 19.36 | 25.79 | 16.15 | 26.11 | 23.27 | 26.49 | 26.57 | 24.45 | 24.93 |
| iLN V3D1-1 | 24.64 | 23.11 | 18.84 | 26.93 | 16.56 | 26.24 | 22.36 | 26.16 | 26.65 | 25.38 | 25.04 |
| iLN V3D1-2 | 25.20 | 23.59 | 19.12 | 27.22 | 16.72 | 26.44 | 22.59 | 26.36 | 26.98 | 25.67 | 25.80 |
| iLN V3D1-3 | 25.34 | 24.43 | 19.06 | 28.20 | 17.28 | 26.64 | 22.83 | 27.12 | 27.07 | 26.26 | 26.73 |
| iLN V3D1-4 | 24.85 | 24.12 | 19.13 | 27.46 | 16.49 | 26.14 | 22.38 | 26.59 | 27.03 | 25.57 | 25.63 |
| iLN V3D1-5 | 24.99 | 23.79 | 19.19 | 27.67 | 16.81 | 25.84 | 22.18 | 26.36 | 26.51 | 25.68 | 25.76 |
| iLN V3D7-1 | 25.05 | 24.73 | 18.59 | 28.23 | 17.44 | 26.40 | 22.74 | 24.47 | 27.34 | 25.63 | 25.95 |
| iLN V3D7-2 | 25.92 | 24.47 | 19.04 | 28.53 | 17.69 | 27.07 | 22.90 | 25.88 | 28.98 | 26.37 | 26.30 |
| iLN V3D7-3 | 25.15 | 24.96 | 18.95 | 28.39 | 17.68 | 26.32 | 22.71 | 25.43 | 28.13 | 26.03 | 26.07 |
| iLN V3D7-4 | 24.86 | 24.23 | 19.53 | 27.87 | 18.67 | 27.66 | 22.73 | 25.35 | 28.49 | 25.25 | 25.80 |

Table S3. Scores obtained from individual software algorithm for 7 reference genes.

|  | **geNorm** | **NormFinder** | **BestKeeper** | **comparative delta-Ct** | **Comprehensive** |
| --- | --- | --- | --- | --- | --- |
| *Actb* | 0.459 | 0.301 | 0.42 | 0.581 | 1.316 |
| *Ywhaz* | 0.48 | 0.355 | 0.346 | 0.604 | 2.213 |
| *Tbp* | 0.459 | 0.453 | 0.424 | 0.645 | 2.449 |
| *Gapdh* | 0.667 | 0.622 | 0.303 | 0.763 | 4.304 |
| *Ubc* | 0.602 | 0.48 | 0.497 | 0.666 | 4.681 |
| *Hprt* | 0.552 | 0.499 | 0.431 | 0.686 | 4.729 |
| *Hmbs* | 0.628 | 0.571 | 0.558 | 0.721 | 6.236 |

Table S4. Scores obtained from individual software algorithm for 4 reference genes.

|  | **geNorm** | **NormFinder** | **BestKeeper** | **comparative delta-Ct** | **Comprehensive** |
| --- | --- | --- | --- | --- | --- |
| *Hmbs* | 0.483 | 0.454 | 0.558 | 0.669 | 1.682 |
| *Hprt* | 0.639 | 0.441 | 0.431 | 0.676 | 1.861 |
| *Ubc* | 0.483 | 0.483 | 0.497 | 0.684 | 2.28 |
| *Tbp* | 0.695 | 0.603 | 0.424 | 0.75 | 2.828 |
